# Fetal Autopsy in Stillbirth: Its Acceptance and Role in Determining the Causes and Risk Factors in North India

**DOI:** 10.7759/cureus.79858

**Published:** 2025-02-28

**Authors:** Anshul Rorvanshi, Rupita Kulshrestha, Ariba Zaidi, Smriti Agrawal, Nuzhat Husain, Gaurav Raj Agarwal, Manodeep Sen, Navbir Pasricha, Neha Agrawal, Deepti Saxena

**Affiliations:** 1 Obstetrics and Gynecology, Dr. Ram Manohar Lohia Institute of Medical Sciences, Lucknow, IND; 2 Pathology, Dr. Ram Manohar Lohia Institute of Medical Sciences, Lucknow, IND; 3 Obstetrics and Gynecology, King George's Medical University, Lucknow, IND; 4 Radiodiagnosis, Dr. Ram Manohar Lohia Institute of Medical Sciences, Lucknow, IND; 5 Microbiology, Dr. Ram Manohar Lohia Institute of Medical Sciences, Lucknow, IND; 6 Anatomy, Dr. Ram Manohar Lohia Institute of Medical Sciences, Lucknow, IND; 7 Reproductive Medicine, Dr. Ram Manohar Lohia Institute of Medical Sciences, Lucknow, IND; 8 Genetics, Sanjay Gandhi Postgraduate Institute of Medical Sciences, Lucknow, IND

**Keywords:** congenital anomalies, fetal autopsy, maternal comorbidities, north india, placental histopathology, stillbirth

## Abstract

Background: Stillbirth is a major global health issue. The fetal autopsy is considered one of the primal diagnostic tools to determine the cause of stillbirth over MRI, ultrasound, and genetic testing. It is often the way to obtain a definitive diagnosis and can be helpful for families who are grieving the loss of a pregnancy. However, its acceptance is limited due to cultural, emotional, and logistical challenges. This study aimed to evaluate the acceptance of fetal autopsy and its role in identifying causative and risk factors for stillbirth in north India.

Materials and methods: This cross-sectional study was performed at a tertiary care center in North India from November 2022 to April 2024. Out of 178 stillbirths among 8500 deliveries, 68 cases met the inclusion criteria, 25 (14%) consented to autopsy, and six (3%) for genetic evaluation. Data were collected on maternal demographics, obstetric history, fetal autopsy findings, placental pathology, and microbiological analysis. The fetal cord blood sample was taken for genetic analysis. Placental tissue, umbilical cord, and fetal oropharyngeal swab samples were taken for culture and histopathological analysis.

Results: In our studied population, the stillbirth incidence rate was 20.94 per 1000 live births. The common maternal comorbidities were preeclampsia (n = 7; 28%) followed by antepartum hemorrhage and diabetes mellitus. The fetal autopsy revealed congenital malformations (n = 9) in 24% of cases, while placental histopathology showed abnormalities in 60%. Microbiological cultures yielded positive results in 36% (n = 9) of cases, with *Staphylococcus *cons (n = 4; 16%) and *Enterococcus faecalis* (n = 2; 8%) being predominant. No genetic abnormality was detected in the six cases that underwent genetic analysis.

Conclusion: The fetal autopsy remains crucial for diagnosing the causes of stillbirth accurately. Effective counseling explaining the benefits of fetal autopsies and genetic testing is essential for improving consent rates and improving pregnancy outcomes. As of now, the acceptance rate for fetal autopsy is less, and for genetic analysis, it is even less, which has a great scope of increment.

## Introduction

Stillbirth is a major global health issue that affects more than 7000 families daily and is linked with emotional, social, as well as economic consequences [1]. As per the Centres for Disease Control and Prevention (CDC), a miscarriage is usually defined as "the loss of a baby before the 20th week of pregnancy, and a stillbirth is the loss of a baby at or after 20 weeks of pregnancy" [2]. Further, stillbirth is described as early (death occurs between 20 and 27 completed pregnancy weeks), late (death occurs between 28 and 36 completed pregnancy weeks), or term (fetal death occurs ≥37 completed pregnancy weeks) [2].

Estimates suggest that there are nearly two million stillbirths every year, one every 16 seconds [1]. Currently, 98% of stillbirths occur in low- to middle-income countries (LMICs), and India has the highest incidence of stillbirths, with an estimated 592,100 fatalities each year, and a WHO-estimated rate of 22 per 1000 live births [3]. The Indian government has produced an ‘Indian Newborn Action Plan’ that includes measures to reduce stillbirths to 10 per 1,000 newborns by 2030 [4]. In 2021, the stillbirth rate (SBR) in India was 13.9 per 1000 live births [4,5]. A reduction in India's SBR would result in the saving of thousands of lives.

The autopsy of a fetus is one of the most accurate diagnostic techniques for diagnosing the cause of death, particularly when anomalies are present [5,6]. For a comprehensive autopsy to be conducted, it adheres to the guidelines and protocols for fetal autopsy [5,6]. These include measurements used to determine the gestational age, foot length, body weight, estimate of the time between death and delivery, the detection of intrinsic defects and developmental issues, and the assessment of any signs of infection [5,6]. The practitioner should transmit the obstetric and relevant medical history to the pathology team and seek any additional tissue samples that may be required for further examination [6,7]. Parental refusal of fetal autopsy is based on cultural, emotional, and religious factors, and doctors frequently struggle to convey that postmortem investigation is an indispensable tool for prenatal diagnosis. Perinatal pathology and fetal autopsy are key components of fetal medicine and clinical genetic services. Few facilities in India are equipped to conduct fetal autopsy. A fetal autopsy has been supported for confirming or correcting prenatal ultrasound data, establishing a definitive diagnosis, and providing genetic counseling and recurrence risk for future pregnancies.

The research gap in the stillbirth cases is identified as nonidentification of the exact cause of stillbirth. Reduced fetoplacental perfusion ultimately leading to fetal death which could be easily identified by using the color Doppler is also missed in the patients. This is one of the preventable causes. Additionally, genetic abnormalities (autosomal recessive disorders resulting from consanguineous marriage and other causes) could be prevented in future pregnancies by doing genetic analysis in stillbirth cases. However, currently in our society, the lack of knowledge, attitude, and practice and sometimes lack of infrastructure leads to the neglect of such investigations. Thus, even preventable causes of fetal death are not identified. To the best of our knowledge, such studies have been sparsely done in the Indian population. This study aims to determine the role of fetal autopsy in reaching a final diagnosis and identify various risk factors (maternal and placental) associated with stillbirth.

## Materials and methods

Study design and setting

In this cross-sectional study, stillbirth cases were selected between November 2022 and April 2024 from the obstetrics and gynecology department of the institution after approval from the Institutional Ethics Committee, Dr. Ram Manohar Lohia Institute of Medical Sciences (RMLIMS), Lucknow (IEC No. 154/22, dated 15.09.2022).

**Figure 1:**
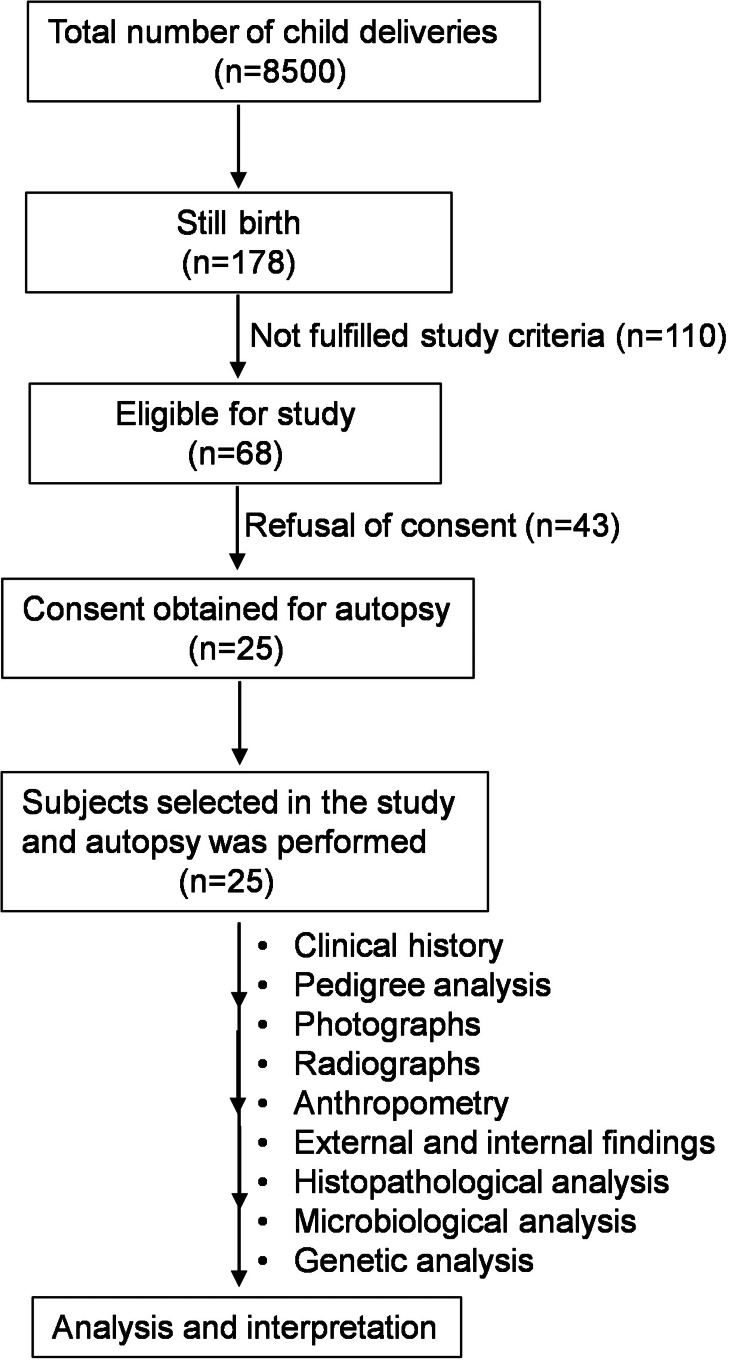
Study flow chart

The study was conducted in collaboration with departments of obstetrics and gynecology, pathology, microbiology, radiology, and anatomy of Dr. RMLIMS, Lucknow, and medical genetics (Sanjay Gandhi Postgraduate Institute of Medical Sciences (SGPGI), Lucknow). A detailed history was taken and recorded as per the predesigned study questionnaire. A detailed study flow chart is depicted in Figure 1.

Convenient sampling was used in this study, and the fetus following delivery was selected. All women who had stillbirths who met the inclusion and exclusion criteria were enrolled in the study. The inclusion criteria for the study were fetal death before or during delivery and who gave consent for autopsy. The exclusion criteria for the study were the family's refusal to consent, macerated fetus (maceration is the softening and breakdown of tissues due to prolonged exposure to amniotic fluid after death suggestive of old intrauterine death), multiple pregnancies, and women with medical termination of pregnancy. An informed written consent was obtained from all the study participants prior to enrolment.

Study participants and sample processing

Subjects were recruited after inclusion and exclusion criteria were satisfied. They were monitored until the fetuses were delivered. Each fetus was subjected to autopsy as soon as possible after the stillbirth to avoid any autolytic changes. The procedure for the autopsy included a photograph, a radiograph of both anterior and lateral views, and an external and internal examination. The placenta, fetal membranes, and umbilical cord were also examined. A small piece of tissue of ~1 cm^3^ was carefully taken from the placenta with a sterile scalpel and forceps, and the tissue was then placed in a sterile solution [7]. Placental swabs and fetal oropharyngeal swabs were sent to the microbiology department to determine infectious causes. Cord blood samples in Ethylenediaminetetraacetic acid (EDTA) vials were sent to the department of medical genetics in SGPGI, Lucknow, for microarray analysis in cases who consented to it. The sections of organs were sent to the department of pathology for histology.

Statistical analysis

To analyze the data, IBM SPSS Statistics for Windows, Version 21 (Released 2012; IBM Corp., Armonk, New York, United States) and MS Excel (Microsoft Corporation, Redmond, Washington, United States) were used. For numbers that could change continuously, we reported the average and how spread out those numbers were. For things that could only be in specific groups, we reported the percentage of things in each group. We used the Chi-square test to see if there were any important differences between groups. We considered a result to be important if the "p-value" was less than 0.05.

## Results

Prevalence of stillbirth

Over 18 months, 8500 deliveries occurred at our center, with 178 stillbirths. Out of these, 68 women met the inclusion criteria, but only 25 (14%) consented for fetal autopsy and six (3%) for genetic evaluation. In 43 cases, a fetal autopsy could not be performed as the fetuses were macerated. In this study, the incidence rate of stillbirth is calculated as 20.94 per 1000 live births.

Baseline characteristics

The mean age of women was 29.44 ± 5.22 years (Table 1). The mean pre-pregnancy weight was 57.24 ± 7.43 kg. The mean height was 1.56 ± 0.07 meters. Out of 25 women, 20 had a BMI between 18 and 24.9 (kg/m^2^) and five women had a BMI between 25 and 29.9 (kg/m^2^). The mean value of systolic blood pressure was 126.80 ± 16.51 mm Hg, and the mean diastolic blood pressure was 80.08 ± 11.98 mmHg. The mean hemoglobin on admission was 10.29 ± 1.33 g/dl. The mean value of random blood sugar was 101.68 ± 25.54 mg/dl. The mean value of HbA1C was 5.54 ± 0.99%. One woman had a history of tobacco addiction and none with history of alcohol intake was found. The majority of women (88%) had gestational ages between 28 and 40 weeks, with equal percentages (44%) in the 28-36 weeks and 37-40 weeks. A total of 17 (68%) cases were unbooked (Table 2), mostly referred from nearby district hospitals and community health centers. Two (8%) women had a previous history of stillbirth, and five (20%) women had a history of previous abortion. With seven (28%) cases, hypertensive disorders of pregnancy (HDP) were the common maternal comorbidity, followed by two (8%) women each with antepartum hemorrhage, type 2 diabetes mellitus (T2DM), and intrahepatic cholestasis of pregnancy.

**Table 1:** Baseline characteristics and comorbidities in study groups SD: standard deviation; BMI: body mass index; SBP: systolic blood pressure; DBP: diastolic blood pressure; RBS: random blood sugar; HbA1C: glycated hemoglobin; DM: diabetes mellitus; GA: gestational age

Baseline characteristics	Subgroup	n = 25	Percentage (%)	Mean ± SD
Age (years)	<20 yrs	1	4	29.44 ± 5.22
	21-30 yrs	15	60	
	31-40 yrs	8	32	
	>40 yrs	1	4	
Pre-pregnancy weight (kg)	-	-	-	57.24 ± 7.43
Height (m)	-	-	-	1.56 ± 0.07
BMI (kg/m^2^)	<18	0	0	23.51 ± 2.62
	18-25	20	80	
	25-29.9	5	20	
Booking status (women with antenatal care)	Booked	8	32	
(Women referred from other centers)	Unbooked	17	68	
Blood pressure (SBP/DBP, mmHg)	-	-	-	126.80 ± 16.51/80.08 ± 11.98
Gestational age	20-27 weeks	3	12	
	28-36 weeks	11	44	
	37-40 weeks	11	44	
Gravida	G1	10	40	
	G2/3	11	44	
	G4/5	4	16	
Parity	P0	14	64	
	P1/2	8	32	
	P3/4	3	12	
Previous abortion	(<20 weeks GA)	5	20	
Previous stillbirth	(>20 weeks GA)	2	8	
Substance abuse	Tobacco	1	4	
Maternal comorbidities	Preeclampsia	7	28	
	Antepartum hemorrhage	2	8	
	Known type 2 diabetes mellitus	2	8	
	Intrahepatic cholestasis	2	8	
	Anaemia (moderate)	1	4	
	Gestational DM	1	4	
	Hepatitis C+	1	4	
	Hypothyroidism	1	4	
	Previous uterine scar	1	4	
	Premature rupture of membranes	1	4	
Hemoglobin at admission (g/dl)	-	-	-	10.29 ± 1.33
RBS (mg/dl)	-	-	-	101.68 ± 25.54
HbA1C	-	-	-	5.54 ± 0.99

**Table 2:** Anthropometric characteristics of fetuses

Fetal characteristics	Category	Number (n)	Percentage (%)
Birth weight	Within 2SD	23	92
	>±2SD	2	8
Crown-rump length (CRL)	Within 2SD	19	76
	>±2SD	6	24
Femur length (FL)	Within 2SD	18	72
	>±2SD	7	28
Head circumference (HC)	Within 2SD	15	60
	>±2SD	10	40
Chest circumference (CC)	Within 2SD	6	24
	>±2SD	19	76
Morphological abnormalities			
Cephalohematoma	Present	5	20
Cyanosed lips	Present	2	8
Subdural hematoma	Present	2	8
Hydrocephalus	Present	1	4
Potter facies	Present	1	4
Periorbital edema	Present	1	4
Lower limb congestion	Present	1	4
External genital malformation	Present	1	4

Fetal anthropometry and morphological abnormalities

Fetal anthropometric parameters and gross morphological abnormalities observed in the autopsy were tabulated in Table 2. On external examination, multiple characteristics were seen. Out of which, cephalohematoma was most common in five (20%) fetuses followed by subdural hematoma in two (8%). Other gross morphological findings in various organ systems are presented in Table 2. Autopsy findings and placental and umbilical cord characteristics associated with stillbirth were recorded in Table 3 as observed.

**Table 3:** Summary of internal fetal autopsy findings, placental, and umbilical cord characteristics

Parameter	Category	No	Percentage
Fetal characteristics			
Proven fetal infections	Present	9	36
Congenital anomaly	Present	6	24
Fetal growth restriction (FGR)	Present	5	20
Fetomaternal hemorrhage and isoimmunization	Absent	0	0
Placental characteristics			
Placental weight	>±2SD	12	48
	Within 2SD	13	52
Signs of infection	Present	2	8
Necrosis	Present	1	4
Ischemia	Absent	0	0
Umbilical cord characteristics			
Cord length	>±2SD	4	16
	Within 2SD	21	84
Appearance	Normal	20	80
	Thrombosed	4	16
	Dark colored	1	4
Location of insertion	Central	24	96
	Marginal	1	4

Fetal autopsy and placental histopathology findings

**Figure 2:**
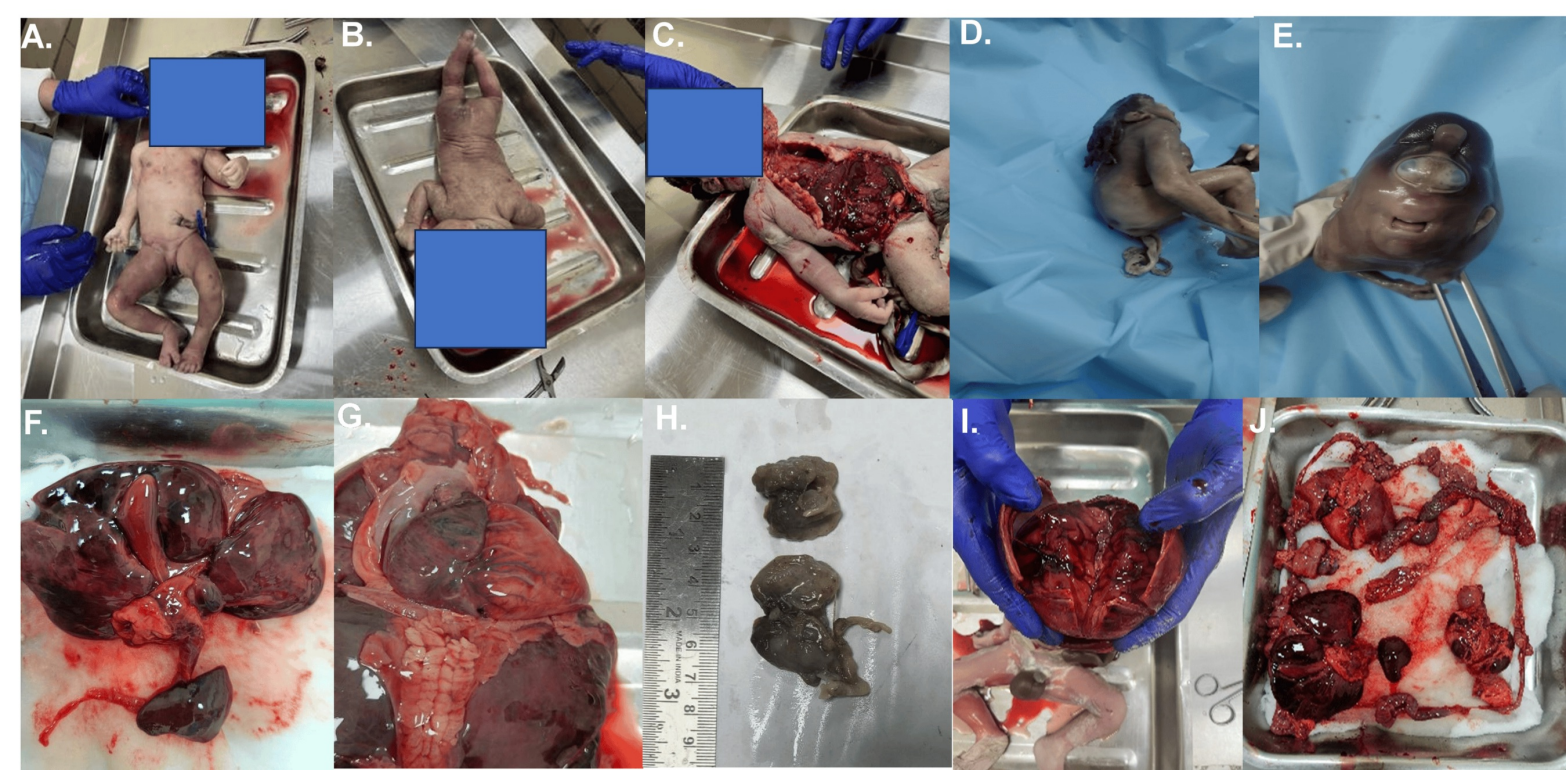
Gross autopsy findings (A) Hydrocephalus with cystic neck swelling (anterior view). (B) Hydrocephalus with cystic neck swelling (posterior view). (C) Anterior midline incision. (D) Anencephaly with spina bifida. (E) Holoprosencephaly with cyclops. (F) Hepatobilliary pancreaticosplenic dissection. (G) Thoracoabdominal dissection. (H) Multicystic dysplastic kidney. (I) Cranial dissection. (J) Dissected multiple organs

**Figure 3:**
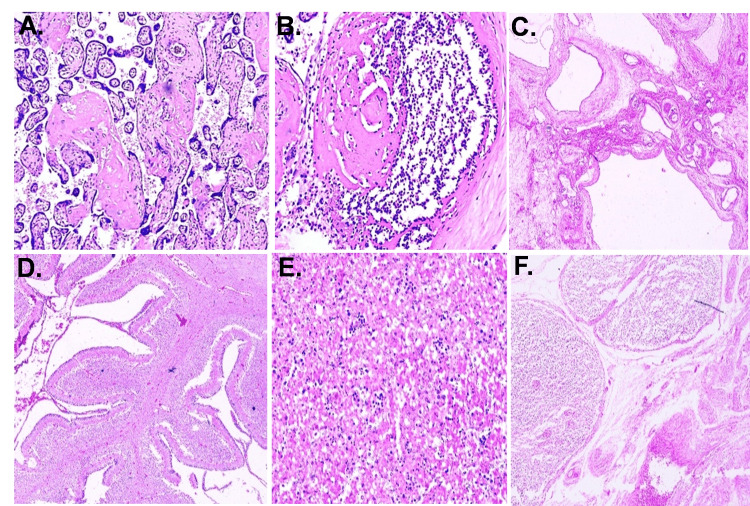
Histopathology of different placentae (A) Placental villi with focal necrosis and increased syncytial knots. (B) Chorioamnionitis with dense inflammatory cells in the placenta. (C) Multicystic dysplastic kidney. (D) Cerebellum. (E) Liver parenchyma showing extramedullary hematopoiesis. (F) Reactive mesenteric lymph node

On histopathological examination of the section of organs, we found congenital malformation in 24% of women (n = 6) (Tables 3, 4). On histopathological examination of placental tissues, we found pathological findings in 60% (15 women), while in 11 women, normal placental morphology was seen (Figures 2, 3).

**Table 4:** Congenital malformations observed in fetal autopsy

Terms	Gross & microscopic findings in the fetus	Gross and microscopic features in the placenta and cord
Congenital malformation, Arnold-Chiari malformation, Dandy-Walker malformation	External findings: the head is enlarged with an edematous neck region, and ribs are compressed on the right side Internal findings: Skull sutures are wide apart, hydrocephalous present with dilated ventricles and microgyria. Orange color pigment deposited over meninges. The right atrium is dilated and contains blood clots. Microscopic findings: meninges are congested. The cortex was thinned out. Foci of lymphomononuclear infiltrate seen with degenerative changes	Gross: Maternal surface was congested with few hemorrhagic areas. The fetal surface was meconium-stained and covered with shiny membranes. Microscopic: stem villi were dilated and had congested blood vessels. Intervillous hemorrhage and calcification in terminal villi with increased formation of syncytial knots were seen as mixed inflammatory infiltrates. Suggestive of maternal vascular malformation with acute chorioamnionitis
Congenital heart anomaly	External findings: unremarkable Internal findings: cephalhematoma, hemorrhagic serous fluid(15 ml) in the thoracic cavity, cardiomegaly present, no valvular heart defect, hepatomegaly for gestational age,20-30 ml serosanguineous fluid in the abdominal cavity. Microscopic findings: focal degenerative changes in the brain, and lungs show congested large- and small-sized vessels, hypertrophic changes in cardiac muscle, congested sinusoids in the liver	Gross: Maternal surface is congested with few hemorrhagic areas. Fetal membranes are seen extending onto the maternal surface. The fetal surface is shiny and covered with shiny membranes. Microscopic: sections show stem villi with dilated and congested blood vessels. Intervillous hemorrhage, calcification in terminal villi with increased formation of syncytial knots and fibrinoid deposits. Suggestive of maternal vascular insufficiency with underlying hypertensive changes
Congenital cystic kidney disease	External findings: features of intrauterine constraints present with compressed limbs, and potter facies. Internal findings: bilateral kidneys are enlarged (right>left), polycystic kidneys. Microscopic findings: renal parenchyma is replaced by numerous cysts lined by cuboidal to flattened epithelium, occasional immature glomeruli	Normal morphology of placenta
Spina bifida	External findings: closed neural tube defect in the thoracolumbar area. Internal findings: closed neural tube defects in the thoracolumbar area	Normal morphology of placenta
Nonimmune hydrops	External findings: generalized edema is seen. Internal findings: subcutaneous edema and fluid were noted in subcutaneous tissue, fluid was noted in the thoracic and abdominal cavity, and pericardial effusion seen	Normal morphology of placenta
Holoprosencephaly with cyclops with proboscis	External findings: holoprosencephaly, cyclopia, short spine, hyperextended neck. Internal findings: short spine, the brain shows monoventricular cavity, fused thalami, fused orbit, single eyeball (synopthalmia) present at the root of the nose	Normal morphology of placenta

Distribution of fetuses on the basis of microbiology findings

**Figure 4:**
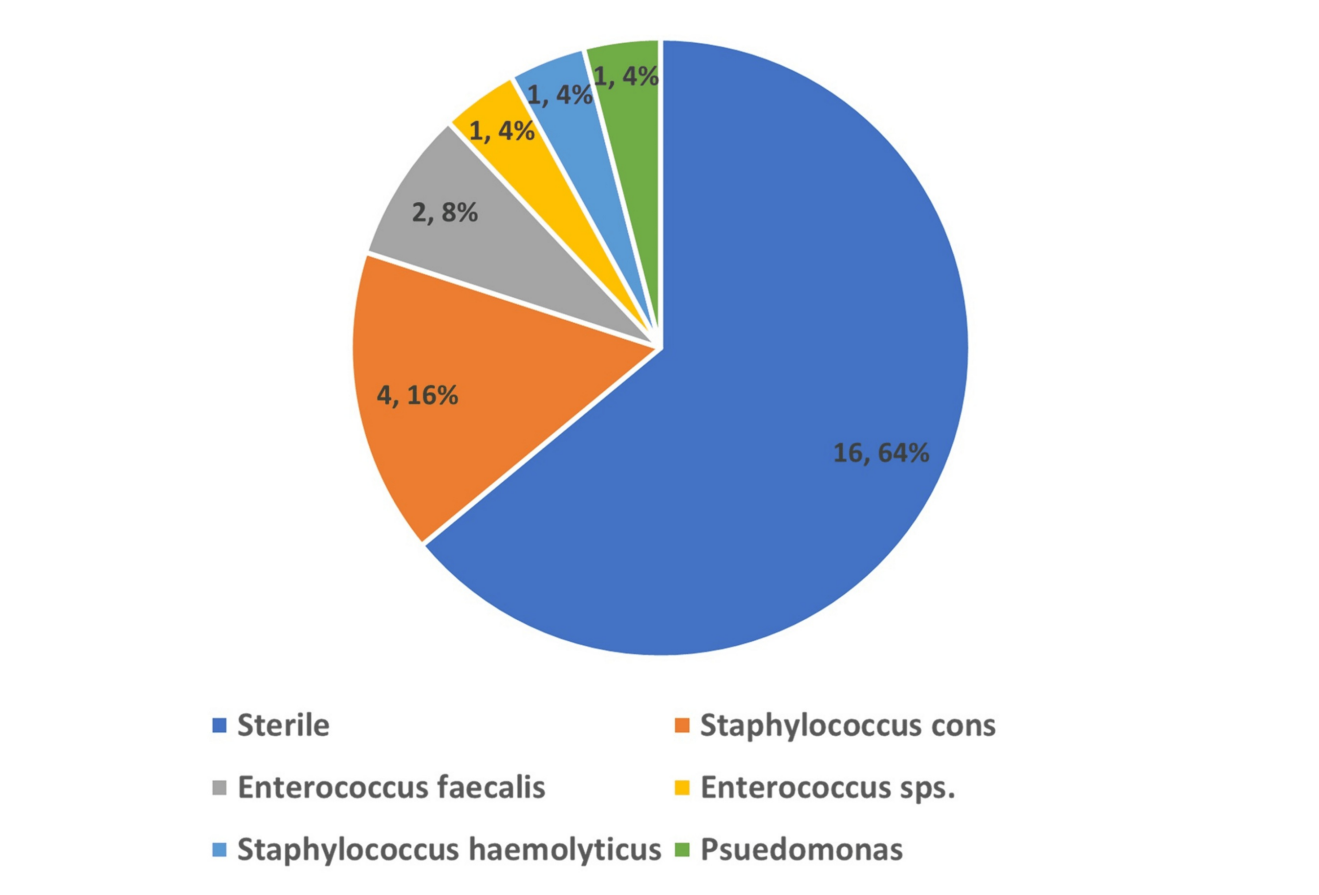
Distribution of women on the basis of swab culture findings

On microbiological culture findings, 16 (64%) cultures were sterile, four (16%) cultures showed growth of* Staphylococcus* cons, two (8%) cultures showed growth of *Enterococcus faecalis*, one (4%) culture showed growth of *Enterococcus *species, one (4%) culture showed growth of *Staphylococcus hemolyticus*, and one (4%) culture showed growth of *Pseudomonas *(Figure 4). 

Association of fetal autopsy findings with placental histopathology

On the association between fetal autopsy findings and placental histopathology findings, there were four (66.7%) women in which both fetal autopsy findings and abnormalities in placental histopathology were present, two (33.3%) women in which fetal autopsy findings were present but no abnormalities in placental histopathology, eight (32%) women in which no fetal autopsy findings and no abnormalities in placental histopathology were seen, and 11 (44%) women in which abnormalities in placental histopathology and no fetal autopsy findings were seen (Table 5).

**Table 5:** Correlation of fetal autopsy findings with placental histopathology

Variables		Fetal autopsy				Statistical analysis
		Congenital malformation		No congenital malformation		
		No. of women	Percentage	No. of women	Percentage	
Placental histopathology	No abnormality	2	8	8	32	p = 0.72
	Abnormalities	4	16	11	44	

Association of microbiological findings with placental histopathology findings and fetal autopsy

It was found that in three (33.3%) women, no abnormality in placental histopathology and growth in microbiology were present; in six (66.7%), women abnormalities and microbiological growth were present; in seven (43.8%), women no abnormalities in placental histopathology and no growth on microbiology were seen; and in nine (56.3%), women abnormalities in placental histopathology and no growth on microbiology were seen (Table 6).

**Table 6:** Correlation of microbiology findings with placental histopathology findings and fetal autopsy

Variables		Microbiology				Statistical analysis
		Growth		Sterile		
		No. of women	Percentage	No. of women	Percentage	
Placental histopathology	No abnormality	3	33.3	7	46.7	0.610
	Abnormalities	6	66.7	8	53.4	
Fetal autopsy	Congenital malformation	3	33.3	3	18.8	0.412
	No congenital malformation	6	66.7	13	81.3	

In the association between microbiological findings and fetal autopsy results, it was observed that three (33.3%) women exhibited both microbiological growth and positive fetal autopsy findings, six (66.7%) women showed microbiological growth without fetal autopsy findings, three (33.7%) women had no microbiological growth but positive fetal autopsy findings, and 13 (81.3%) women presented neither autopsy findings nor microbiological growth (Table 6).

Genetic analysis

No chromosomal anomalies (autosomal aneuploidies: trisomy 13,18,21; sex chromosomal aneuploidies: monosomy X, XXY, XYY, XXX; euploidy: triploidy, duplications, deletions) were noted in any of the women. For one woman in which nonimmune hydrops were seen, a parvovirus B 19 polymerase chain reaction (PCR) was also done, and it showed a negative result. Out of six women, genetic abnormality is not detected in any women.

## Discussion

Fetal autopsy remains the gold standard for understanding the cause of intrauterine fetal death (IUFD), yet the correlation between ultrasound (USG) findings and autopsy outcomes is inconsistent [8]. Faye-Petersen et al. [9] concluded that pathologists working with clinical experts could elucidate the cause of death in 94% of cases. Several studies highlighted that fetal abnormalities, including congenital anomalies, infections, nonimmune hydrops, and malnutrition, account for 25%-40% of stillbirths [10]. Investigators noted that autopsy and placental examination could identify the cause of death in 57.9% of cases, with placental insufficiency more common in the early third trimester and umbilical cord complications appearing closer to term [11].

The International Classification of Diseases (ICD-10) includes codes for stillbirths but does not fully recognize the underlying causes [12]. In developing countries like India, where congenital birth defects contribute significantly to neonatal mortality, improving the understanding of stillbirth causes is vital. A comprehensive postmortem investigation of stillbirth is necessary for determining the cause of death, guiding counseling for parents, and informing subsequent pregnancy management.

This study, conducted on 25 women, reported a mean maternal age of 29.44 years, consistent with findings from other studies [12,13]. A total of 4% of the women had a history of tobacco addiction, and no one reported alcohol intake. A total of 32% were booked antenatal cases, and 20% had a history of previous abortion. Maternal age, gravida status, and gestational age at the time of stillbirth mirrored findings from other studies as well [14-16], with the majority of women being primigravida or nulliparous.

Maternal comorbidities such as pre-eclampsia, antepartum hemorrhage, type 2 diabetes, and intrahepatic cholestasis of pregnancy were observed in 28%, 8%, 8%, and 8% of cases, respectively. These findings are in agreement with studies by Rasheed et al. [13] and Sade et al. [15], who also identified hypertension, diabetes, and other comorbidities as significant contributors to stillbirth. Hypertensive disorders and diabetes are recognized as among the most common maternal conditions linked to stillbirth as in this study too [10,16]. Preventable causes like trauma, infection, and umbilical cord accidents account for the majority of stillbirths, underscoring the Importance of adequate prenatal care and timely interventions.

In this study, fetal infections were identified in 32% of cases, while congenital fetal defects were the second most common condition, found in 24% of cases. Other fetal conditions, including fetal growth restrictions (20%), nonimmune hydrops, etc., were identified in 4% of cases. These findings align with previous researches [8,15-16], which identified congenital anomalies and infections as major fetal contributors to stillbirth. Notably, this study also found no cases of fetomaternal hemorrhage or isoimmunization consistent with other studies [17]. Literature suggests that when closely related people have children (consanguineous unions), there's a higher chance of the children having inherited diseases [18]. While, in this study, none of the cases gave a positive history of consanguinity, so the role of it as studied in cases of recessive chromosomal disorders could not be studied here [18].

In this study, it was found that placental pathology played a significant role in stillbirth, with 52% of placentas within normal weight limits, while 48% exhibited abnormalities. A study by Korteweg et al. [19] emphasized placental bed pathologies as a dominant cause of fetal death, a finding also reflected in the present study, which showed that 38.5% of cases exhibited placental bed pathologies. Placental necrosis, infection, and other abnormal findings were observed more frequently in this study compared to others [12,18-20]. Placental insufficiency and other placental abnormalities are known to contribute significantly to fetal death [11,14,18-20].

In this study, umbilical cord abnormalities were noted in a few cases, with 16% of cords having abnormalities in length and appearance. This is consistent with studies that report umbilical cord anomalies, such as abnormal insertion and thrombosis, as contributing factors in some stillbirths [12,14,21]. However, the present study found no significant association between umbilical cord abnormalities and stillbirth, contrasting with some other studies that identified such abnormalities as major contributors to stillbirth [11,20]. In terms of histopathological examination, congenital malformations were identified in 24% of cases, with 60% of placental samples showing abnormalities. Histopathological findings were largely consistent with clinical conditions, helping pinpoint potential causes of stillbirth, though no significant correlation between microbiological findings and placental abnormalities was observed. Only 12% of cultures grew pathogenic organisms such as *Staphylococcus *species and *Pseudomonas*, suggesting that infection was not a dominant cause of stillbirth in this cohort.

Overall, this study emphasizes the importance of integrating fetal autopsy, placental examination, and clinical data to uncover the causes of stillbirth. These results align with findings from other studies, which emphasize the value of autopsy and placental examination in understanding stillbirth mechanisms and improving future pregnancy outcomes. In spite of parental counseling in such cases, the acceptance rate of the fetal autopsy was only 14% and that for genetic analysis was even less (4%). Parental refusal of fetal autopsy is probably based on cultural, emotional, and religious factors, and doctors frequently struggle to convey that postmortem investigation is an indispensable tool for prenatal diagnosis.

The study’s strength lies in its use of fetal autopsy reports, ensuring a high level of precision in determining the causes of stillbirth, reducing the subjectivity typically associated with clinical diagnoses. This approach ensures a high degree of certainty in distinguishing between causal and noncausal factors. The uniqueness of this study lies in the fact that maternal, placental, umbilical cord-related, microbiological, and genetic factors have been studied with accuracy. Our findings highlight the multifactorial nature of stillbirth, with placental pathology, maternal comorbidities, congenital malformations, and fetal growth restriction emerging as key factors. The strong association between placental abnormalities and adverse pregnancy outcomes suggests that improved placental monitoring during pregnancy could aid in the early detection of complications, potentially preventing stillbirth. Further, the lack of genetic abnormalities in our cohort calls for a more targeted approach to genetic screening, perhaps focusing on high-risk populations.

The sample size of our study was limited to 25 cases of stillbirth where fetal autopsies were performed, and only six cases underwent genetic analysis. This small sample size reduces the generalizability. In addition to this, this study was conducted at a single institution, limiting the external validity of the findings. The low consent rate for autopsy also points to the need for better education and communication strategies to address cultural, emotional, and religious barriers surrounding postmortem examinations. Establishing trust and understanding with grieving families is crucial in promoting the acceptance of autopsy as a diagnostic tool. Future multicentric studies with larger sample sizes and more comprehensive genetic and microbiological testing are needed to further validate and expand upon our results.

## Conclusions

Fetal autopsy is a primal yet underutilized tool in determining the causes and risk factors related to stillbirth. In this study, while fetal autopsies provided definitive diagnoses in a major proportion of cases, their acceptance remained low, primarily due to cultural and emotional barriers. Additionally, the identification of congenital malformations, placental abnormalities, and infectious etiologies through postmortem evaluation highlights its invaluable role in guiding clinical decisions and future pregnancy management. Integration of fetal autopsies with placental histopathology and microbiological investigations can refine our understanding of stillbirth, aiding in preventive strategies. Moreover, the limited uptake of genetic analyses underscores the need for enhanced counseling and awareness programs to improve consent rates. Our efforts should focus on addressing sociocultural concerns and enhancing parental education regarding the benefits of fetal autopsies and genetic testing to improve perinatal outcomes.

## Appendices

**Table 7:** Study questionnaire and data collection sheet USG: ultrasound; PPROM: preterm premature rupture of membranes; PROM: premature rupture of membranes; VDRL: venereal disease research laboratory; LMP: last menstrual period; Hb: hemoglobin; GTT: glucose tolerance test; GCT: glucose challenge test; HbA1c: glycated hemoglobin

Case record form and data collection sheet					
Verbal autopsy □					
Autopsy □					
Genetic evaluation □					
Patient’s hospital registration number					
Date of enrolment					
Patient name					
S.N.					
1	Patient details				
	Maternal age (years)				
	Address/residence				
	Religion				
	Tribe				
	Pre-pregnancy weight (kg)				
	Height (m)				
	BMI (kg/m^2^)				
	BP (mmHg): an admission		Treatment given, if any:		
	GTT /GCT		If deranged	HbA1c	
				Bood sugar fasting	
				Postprandial	
	HB		Treatment given, if any:		
	Blood group				
	RH factor	Positive □			
		Negative □	Any previous anti-D immunization	Yes □	
				No □	
	Education level	No formal education	□		
		Primary	□		
		Secondary	□		
		College/university	□		
	Marital status	Single □	Married □	Other □	
	Employment status	Not employed □	Employed (specify) □		
	Tobacco use	Yes □		No □	
	Alcohol use	Yes □		No □	
	Maternal comorbidity	Chronic hypertension	□		
		Severe preeclampsia/ eclampsia	□		
		Heart disease	□		
		Diabetes mellitus 2	□		
		HIV/AIDS	□		
		Previous scars	□		
		PPROM/PROM	□		
		Thyroid disorders	□		
		VDRL	□		
		Asthma	□		
		Renal disease	□		
		Intrahepatic cholestasis	□		
		Other	□		
	Previous stillbirth	Yes	□	No	□
	Previous abortion	Induced	□	Spontaneous	□
	Placenta location	Normal		□	
		Previa		□	
		Abruption		□	
	Gravidity				
	Parity				
	Gestation age (at delivery by LMP)				
	Gestation age (at delivery by 1st ultrasound)				
	Booked/unbooked	Booked	□	Unbooked	□
	Mode of delivery	Vaginal delivery	□	Caesarean section	□
	Antepartum hemorrhage				
	Hb level at delivery				
	Gross examination of the placenta and membrane	Dimensions			
		Overt presence of infection			
		Ischemia			
		Necrosis			
	Cord length				
	Cord appearance	Insertion	Normal	□	
			Marginal	□	
		Cord knot			
		Velamentous cord			
		Others (Specify)			
	Cord location	Normal	□		
		Nuchal cord	□		
		Cord presentation	□		
		Cord prolapsed	□		
	Birth weight				
2	Pedigree				
3	USG findings				
4	Photograph				
5	Radiograph	Anterior view		Lateral view	
6	Anthropometry				
		Parameters	Measurement	Centile	
	Weight				
	Total length				
	Crown-rump length				
	Foot length				
	Head circumference				
	Chest circumference				
	Other important measurements				
7	External findings				
	External malformations				
	Skull				
	Eyes				
	Ears				
	Nose				
	Plate				
	Lips				
	Upper limbs				
	Hands				
	Lower limbs				
	Feet				
	Chest				
	Abdominal wall				
	External genitalia				
	Umbilical cord				
	Placenta				
	Anal opening				
	Others				
8	Internal findings				
	Brain				
	Ventricles				
	Corpus callosum				
	Cerebellum				
	Spine				
	Neck structures				
	Lungs				
	Heart				
	Major vessels				
	Diaphragm				
	Stomach				
	Liver				
	Kidneys				
	Adrenals				
	Ureters				
	Bladder				
	Internal genitalia				
	Intestines				
	Rectum				
	Other findings				
9	Histopathology				
10	Microbiological analysis				
11	Genetic evaluation				
12	Provisional/final diagnosis				
13	Counseling and advice				
